# Development and Validation of a Novel Gene Signature for Predicting the Prognosis by Identifying m5C Modification Subtypes of Cervical Cancer

**DOI:** 10.3389/fgene.2021.733715

**Published:** 2021-09-22

**Authors:** Jing Yu, Lei-Lei Liang, Jing Liu, Ting-Ting Liu, Jian Li, Lin Xiu, Jia Zeng, Tian-Tian Wang, Di Wang, Li-Jun Liang, Da-Wei Xie, Ding-Xiong Chen, Ju-Sheng An, Ling-Ying Wu

**Affiliations:** ^1^ Department of Gynecologic Oncology, National Cancer Center/National Clinical Research Center for Cancer/Cancer Hospital, Chinese Academy of Medical Sciences and Peking Union Medical College, Beijing, China; ^2^ Department of Blood Grouping, Beijing Red Cross Blood Center, Beijing, China; ^3^ State Key Laboratory of Molecular Oncology, National Cancer Center/National Clinical Research Center for Cancer/Cancer Hospital, Chinese Academy of Medical Sciences and Peking Union Medical College, Beijing, China

**Keywords:** cervical cancer, m5C modification, signature, prognosis, TCGA

## Abstract

**Background:** 5-Methylcytidine (m5C) is the most common RNA modification and plays an important role in multiple tumors including cervical cancer (CC). We aimed to develop a novel gene signature by identifying m5C modification subtypes of CC to better predict the prognosis of patients.

**Methods:** We obtained the expression of 13 m5C regulatory factors from The Cancer Genome Atlas (TCGA all set, 257 patients) to determine m5C modification subtypes by the “nonnegative matrix factorization” (NMF). Then the “limma” package was used to identify differentially expressed genes (DEGs) between different subtypes. According to these DEGs, we performed Cox regression and Kaplan-Meier (KM) survival analysis to establish a novel gene signature in TCGA training set (128 patients). We also verified the risk prediction effect of gene signature in TCGA test set (129 patients), TCGA all set (257 patients) and GSE44001 (300 patients). Furthermore, a nomogram including this gene signature and clinicopathological parameters was established to predict the individual survival rate. Finally, the expression and function of these signature genes were explored by qRT-PCR, immunohistochemistry (IHC) and proliferation, colony formation, migration and invasion assays.

**Results:** Based on consistent clustering of 13 m5C-modified genes, CC was divided into two subtypes (C1 and C2) and the C1 subtype had a worse prognosis. The 4-gene signature comprising FNDC3A, VEGFA, OPN3 and CPE was constructed. In TCGA training set and three validation sets, we found the prognosis of patients in the low-risk group was much better than that in the high-risk group. A nomogram incorporating the gene signature and T stage was constructed, and the calibration plot suggested that it could accurately predict the survival rate. The expression levels of FNDC3A, VEGFA, OPN3 and CPE were all high in cervical cancer tissues. Downregulation of FNDC3A, VEGFA or CPE expression suppressed the proliferation, migration and invasion of SiHa cells.

**Conclusions:** Two m5C modification subtypes of CC were identified and then a 4-gene signature was established, which provide new feasible methods for clinical risk assessment and targeted therapies for CC.

## Introduction

It is estimated that 310,000 people die of CC every year worldwide, CC is fourth most common cause of cancer-related death in women and constitutes a major public health problem (Bray et al., 2018; Arbyn et al., 2020). Every 2 min, one woman dies of CC (Knaul et al., 2019). Human papillomavirus (HPV) infection is a major risk factor for CC, with approximately 90% of cases occurring in low-income and middle-income countries lacking organized screening and HPV vaccination programs (Lagheden et al., 2018; Cohen et al., 2019). For underdeveloped countries, the scarcity of resources and infrastructure limits disease prevention and treatment plans, even no prevention and treatment options are available in some areas. Patients with CC often have social difficulties, constipation, diarrhea, severe lymphedema, menopausal symptoms and major financial problems (Cohen et al., 2019). So it is necessary to improve the diagnosis and treatment methods which need to show cost-effective patient-centered improvements compared with the current strategies (Seol et al., 2014). At present, the conventional treatment of CC includes radiotherapy, chemotherapy and surgery. However, patients with advanced-stage disease are prone to resistance to radiotherapy and chemotherapy. Although immunotherapy is becoming an effective adjuvant therapy, most therapeutic vaccines are still in the early experimental stage (Alldredge and Tewari, 2016). Therefore, it is urgent to determine new prognostic indicators and treatment options to improve the survival rate of patients with CC.

In recent years, the epigenetic modification of RNA has become a focus of research; the dynamic regulation and disturbance of these RNA modifications are also significantly related to the occurrence, maintenance and progression of tumors (Han et al., 2020). RNA contains several dynamic modifications, including N6- methyladenosine, 5-methylcytosine and N7-methylguanosine (Schumann et al., 2020; Song et al., 2020; Tang et al., 2021). m5C existing in mRNAs, tRNAs, rRNAs and ncRNAs, is involved in RNA stability and translation efficiency (Squires et al., 2012). Currently, 13 regulatory factors are involved in the process of m5C methylation. The dynamic modification of m5C is regulated by writers (methyltransferase), readers (binding protein), and erasers (demethylase) (Chen et al., 2019). “Writer” complexes, including NOP2, NSUN2, NSUN3, NSUN4, NSUN5, NSUN6, NSUN7, DNMT1, DNMT3A, DNMT3B and TRDMT1, increase methylation at the RNA C5 site (Bohnsack et al., 2019). “Reader” protein ALYREF could recognize and bind to methylated RNA, and “Eraser” protein TET2 could change the modification of m5C by demethylation (Yang et al., 2017). However, the function and molecular mechanism of m5C-related regulators in CC remain unknown.

In this study, we classified CC subtypes according to these 13 currently reported m5C regulatory factors, further explored DEGs in different CC subtypes, and finally identified a 4-gene signature that could predict the prognosis of CC patients.

## Materials and Methods

### Data Download and Preprocessing

Patients with no survival time available and follow-up time of less than 1 month or more than 120 months were excluded, then mRNA data and clinical information of 257 CC patients were downloaded from the TCGA database. The clinical statistical information of the TCGA all set is shown in Additional file 1: Supplementary Table S1. Another dataset GSE44001 consisting of 300 CC patient with associated prognostic information was obtained from the Gene Expression Omnibus (GEO) database.

### Determination of m5C Modification Subtype

First, we extracted 13 m5C regulatory factors from the TCGA expression matrix. Based on consistent clustering of these 13 genes, 257 CC samples were clustered by the “NMF” method, which was used to select the standard “brunet” option for 50 iterations. The number of clusters k was set at 2 to 10 and the average contour width of the common member matrix was determined by the “NMF” package. The minimum member of each subclass was set to 10. According to cophenetic, rss and silhouette, the optimal number of clusters was determined. KM analysis was used to analyze the difference in prognosis between different subtypes of the patients using the “survival” package and heatmaps were drawn using the “pheatmap” package.

### Assessment of Immune Infiltration

In order to identify the immune infiltration differences between different m5C modification subtypes, we used “MCPcounter” package to evaluate the score of 10 immune cells and the score of 28 immune cells were evaluated by the “single sample Gene set enrichment analysis (ssGSEA)” method in the “gene set variation analysis (GSVA)" package (Charoentong et al., 2017). Besides, we analyzed the mRNA level differences of 13 m5c-related genes between different subtypes.

### Identification and GO/KEGG Annotation of m5c Subtype-Related Differentially Expressed Genes

The “limma” package was used to calculate the DEGs between different m5C modification subtypes, and the filter was applied according to the thresholds FDR <0.05 and |log_2_FC| > log_2_ (1.5). Furthermore, 601 upregulated DEGs and 113 downregulated DEGs were analyzed by the “WebGestalt” package for Gene Ontology (GO) and Kyoto Encyclopedia of Genes and Genomes (KEGG) annotation.

### Construction of a Novel Gene Signature Based on m5c Subtype-Related DEGs

Under the premise there is no preference in the distribution of clinical characteristics of the grouped samples, a total of 257 patients in TCGA all set were randomly divided into training set (*n* = 128) and test set (*n* = 129). The TCGA training set and TCGA test set were evaluated by Chi-square test, the sample information is shown in Additional file 1: Supplementary Table S2. In addition, we also used the TCGA all set (*n* = 257) and GSE44001 set (*n* = 300) as validation set for subsequent verification. In the training set, a univariate Cox regression analysis was conducted by the “survival coxph function” package using the 714 DEGs and survival data, *p* < 0.01 was selected as the threshold for filtering. Next, we used the “glmnet” package for the least absolute shrinkage and selection operator (LASSO) regression to further compress the screened genes to reduce the number of genes, and finally a novel gene signature was established. LASSO retains the advantages of subset shrinkage, and is a biased estimate for processing data with multicollinearity. Based on the LASSO regression results, we developed a prognostic risk score formula, which was calculated as follows:

Risk score (patient)= Σi Coefficient(mRNAi) × Expression (mRNAi)

### Validation of the Gene Signature

We calculated the risk score of each sample depending on the signature gene and drew the risk score distribution of the sample in the TCGA training set. Furthermore, we used the “timeROC” package to perform receiver operating characteristic (ROC) analysis to explore the prediction accuracy of 1 year, 3 years and 5 years survival rates. Finally, we calculated the risk score and divided the samples with risk score greater than zero into the high-risk group and samples with risk score less than zero into the low-risk group to draw KM curves. To determine the robustness of the signature, we used the same coefficient to perform the same analysis used for the TCGA test set, TCGA all set and external validation data set GSE44001.

### Gene Set Variation Analysis

We selected the corresponding gene expression profiles of these samples for ssGSEA via the “GSVA” R package to observe the relationship between the risk score and the KEGG pathway. We calculated the score of each sample in different KEGG pathway and obtained the ssGSEA score of each sample. Next, we calculated the correlation between these pathways and the risk score and selected pathways with a correlation greater than 0.4. Finally, the top 18 KEGG pathways were selected and clustered according to their enrichment score.

### Univariate and Multivariate Cox Analysis of the Signature and Construction of a Nomogram

To identify the independence of the gene signature in clinical parameters, we used univariate and multivariate Cox regression to analyze the hazard ratio (HR), 95% confidence interval (CI) of HR and *p* value in the TCGA all set. We systematically analyzed the clinical information of TCGA patient records, including age, T stage, N stage, FIGO stage, grade, chemotherapy and risk score. According to the results of univariate and multivariate Cox analyses, we constructed a nomogram with the T stage and risk score for predicting survival outcomes (1 year, 3 years and 5 years). Then we performed the calibration curve by the “rms” package to determine the consistency between the actual survival rates and the nomogram-predicted rates. In order to evaluate the reliability of the nomogram, we performed DCA (decision curve analysis) using the “rmda” package. DCA analysis is a method that can assess whether the nomogram improves clinical decision-making. This method can tell us whether it is beneficial to use the model to make clinical decisions, or which of the two models will lead to better decisions.

### Tissue Specimens

Fresh adjacent normal tissues and CC tissues were obtained from the Chinese Academy of Medical Sciences and the CAMS & PUMC Medical College. All patients were not treated preoperatively and signed informed consent forms provided by the Cancer Hospital, CAMS & PUMC. The normal surgical margin tissue and the morphology of the primary tumor area were immediately excised from each patient by an experienced pathologist and stored in liquid nitrogen. The study was approved by the Ethics Committee of the Cancer Institute (Hospital), CAMS & PUMC (20/207–2,403).

### Cell Culture and Transfection

The human CC cell line SiHa was provided by the Cell Resource Center, IBMS, CAMS/PUMC. The cell lines were cultured in DMEM medium supplemented with 10% fetal bovine serum (Invitrogen, San Diego, CA) at 37 °C and 5% CO2 in a humidified incubator. Human specific siRNA sequences are shown in Additional file 1: Supplementary Table S3. The transfection method was described in our previous article (Cao et al., 2018).

### Real-Time Quantitative Polymerase Chain Reaction

Total RNA was extracted using RNApure Tissue & Cell Kit (Cwbiotech, Beijing, China). Isolated RNA was used as a template for reverse transcription reactions using HiFiScript cDNA Synthesis Kit (Cwbiotech, Beijing, China). Real-time quantitative PCR analysis was performed using SYBR^®^ Fast qPCR Mix (TaKaRa, Shiga, Japan) and a CFX96 Real-Time System (Bio-Rad, California, United States of America). The primer sequences are shown in Additional file 1: Supplementary Table S4. GAPDH served as the internal control.

### Western Blotting and Immunohistochemistry Analysis

Western blotting and IHC analysis were performed as described previously (Cao et al., 2018). The antibodies used were as follows: anti-FNDC3A antibody (Abcam, Cambridge, United Kingdom), anti-VEGFA antibody (Proteintech, Wuhan, China), anti-OPN3 antibody (Affinity Biosciences, Cincinnati, United States), and anti-CPE antibody (Proteintech, Wuhan, China). The IHC quantization analysis was calculated by ImageJ software and statistically analyzed in three random fields. Data are shown as mean ± SEM.

### Cell Viability Assays

Cells were inoculated into 96-well plates at a concentration of 2000 cells per well. According to the manufacturer’s directions, cell viability was determined by the Cell Counting Kit-8 (CCK-8, Dojindo, Japan). The absorbance was measured at 450 nm by an automatic microplate reader (BioTek, Winooski, United States). Measurements were taken every 24 h for seven consecutive days.

### Colony Formation Assay

SiHa cells treated with siRNA were plated in 6-well plates at a density of 500 cells per well. After overnight incubation, the cells were cultured for 14 days to form colonies, fixed with methanol and stained with crystal violet. The data represent the mean ± SD of three independent experiments.

### Cell Migration and Invasion Assays

700 μL DMEM medium supplemented with 20% serum was added to the lower chamber of the Transwell plates, and 1×10^5^ suspended cells were added to the upper compartment. For the invasion experiment, 50 μL Matrigel was added to the membrane of the upper chamber. The Transwell plates were incubated in a carbon dioxide incubator for 16 h in the migration experiment and for 24 h in the invasion experiment. Then, we removed the chamber, washed the cells once with PBS, fixed the cells with solution (methanol: acetone = 1:1) for 30 min, and then stained them with 0.5% crystal violet for 30 min. The chamber was washed with PBS, and then the upper cells were carefully removed, sealed with neutral gum and photographed for counting.

### Statistical Analysis

R software 3.5.3 and SPSS 22.0 software (SPSS Inc. Chicago, United States) were used for all statistical analyses. *p* < 0.05 was taken as the probability value to establish statistical significance. Chi-square test was used for statistics of multiple categories, Student’s t-test was used to determine the significance of differences between two groups, and ANOVA was used for comparisons among more than two groups.

## Results

### Determination of m5C Modification Subtype

**FIGURE 1:**
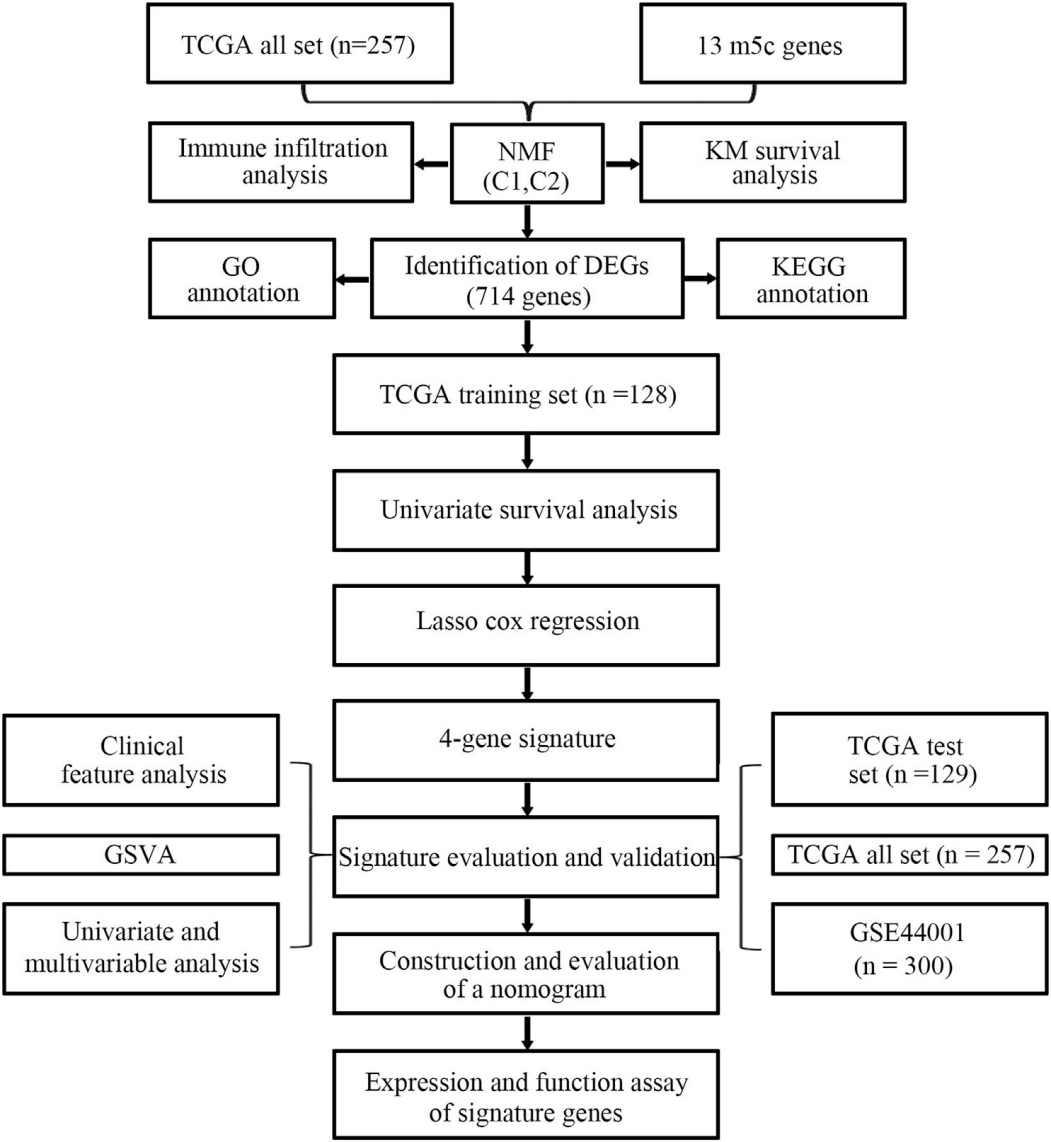
Flow chart for the research.

To clearly illustrate the process of our research, a flow chart is shown in Figure 1.

**FIGURE 2:**
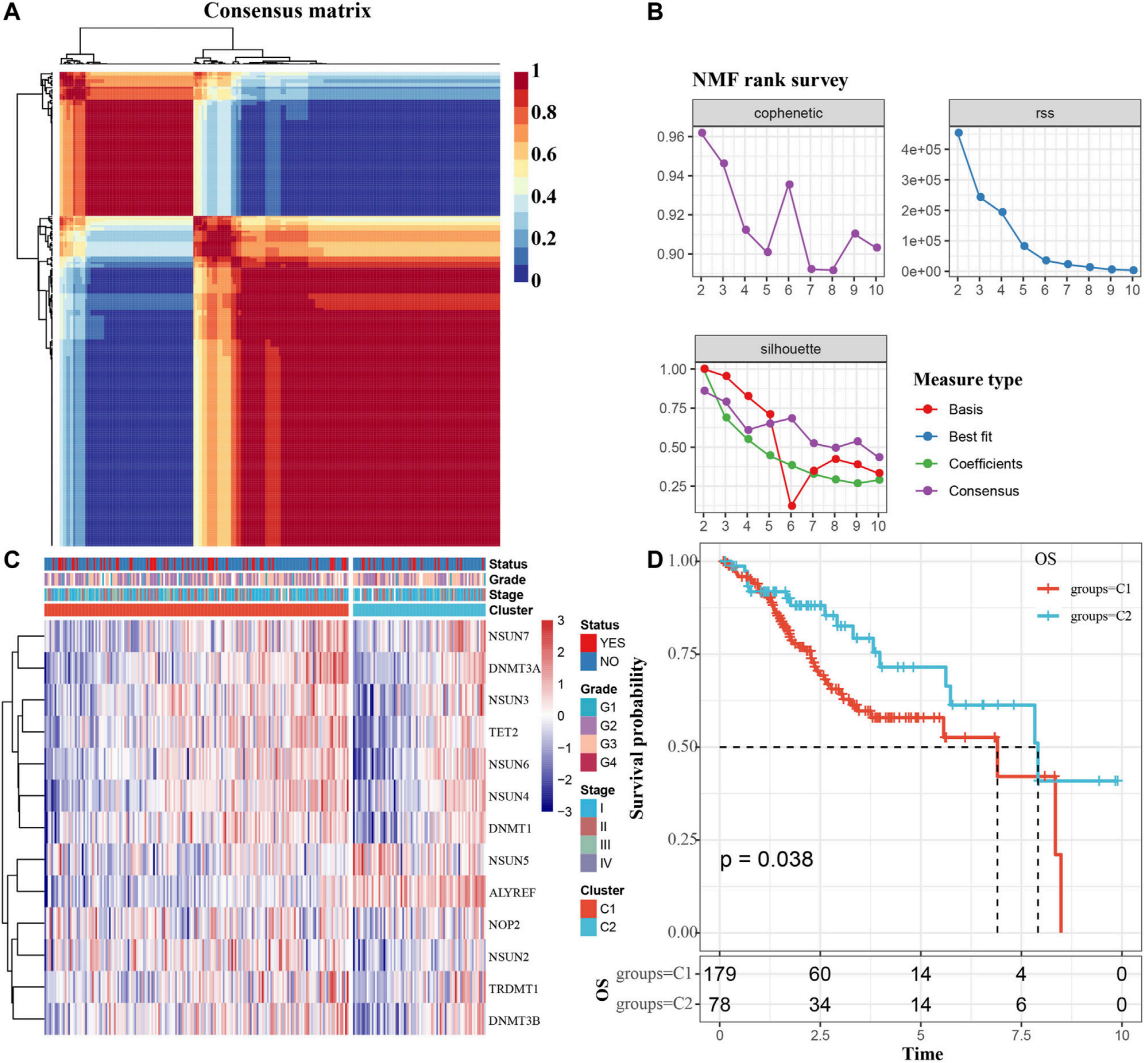
Determination of m5C modification subtype. **(A)** Consensus map for NMF clustering. **(B)** The cophenetic, rss and silhouette distribution when the number of clusters k was set as 2 to 10. The cophenetic correlation is used to reflect the stability of cluster obtained from NMF, we select k value where the magnitude of the cophenetic correlation coefficient begins to fall. The “rss” refers to residual sum of squares, a smaller value represents the effect of model clustering. Silhouette is used to study the distance between clusters of clustering results, When the silhouette value is closer to 1, the clustering is more reasonable. According to cophenetic, rss and silhouette, the optimal number of clusters was two under comprehensive consideration. **(C)** m5c methylation-related gene cluster maps. **(D)** KM survival curves of m5C modification subtypes. Time: years.

We extracted mRNA levels of 13 m5C regulatory factors from the expression matrix of the TCGA. Then, 257 CC samples were clustered by “NMF” package. The optimal number of clusters was determined according to cophenetic, rss and silhouette analyses, the optimal number of clusters was 2 (Figures 2A,B). The expression levels of m5C methylation-related genes in the C1 and C2 subtypes were significantly different (Figure 2C). The KM curve revealed that overall survival (OS) rates of the C1 and C2 subtypes were significantly different (*p* < 0.05), and the prognosis of the C1 group was worse than that of the C2 group (Figure 2D).

### Immune Infiltration Analysis of m5C Modification Subtype

**FIGURE 3:**
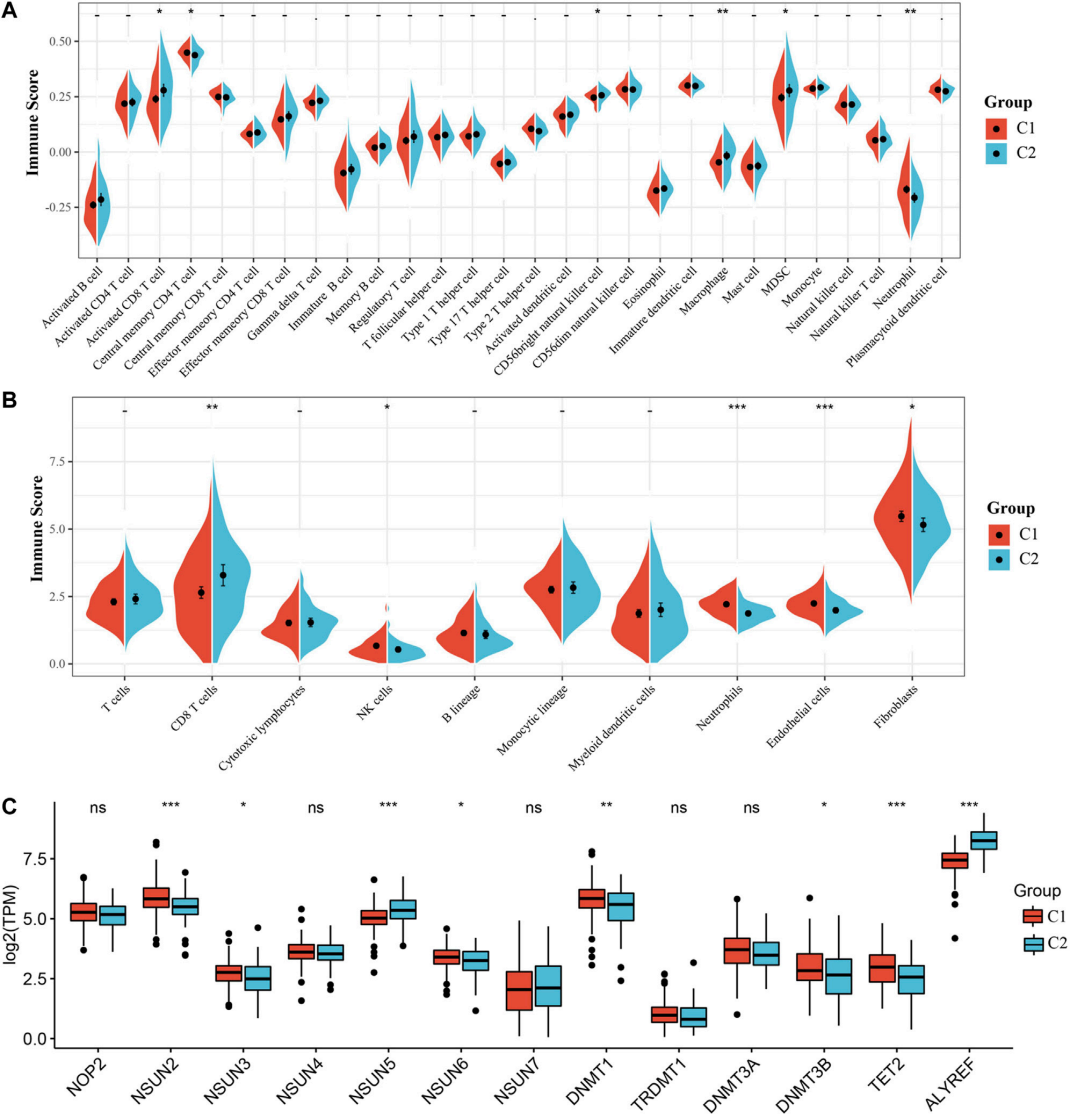
Immune infiltration analysis of the m5C modification subtype. **(A)** Comparison of ssGSEA immune score between C1 and C2 subtypes in the TCGA all set. **(B)** Comparison of MCPcounter immune score between two subtypes in the TCGA all set. **(C)** Expression differences of 13 genes related to m5C modification between two subtypes.

Due to the significant difference in the prognosis of CC patients with two m5C modification subtypes, we next explored the difference in immune cell infiltration between C1 and C2 subtypes. The ssGSEA score suggested that levels of activated CD8 T cells, central memory CD4 T cells, CD56 bright natural killer cells, macrophages, MDSCs and neutrophils were markedly different between two subtypes, and the MCPcounter score indicated that the infiltration levels of CD8 T cells, NK cells, neutrophils, endothelial cells and fibroblasts were significantly different (Figures 3A,B). Furthermore, we also analyzed the expression of 13 genes between two subtypes. The expression of eight genes in C1 and C2 subtypes was substantially different; but no difference was found in NOP2, NSUN4, NSUN7, TRDMT1 and DNMT3A expression (Figure 3C). The above results indicated that there are significant differences in the immune infiltration of C1 and C2 subtypes, while the expression of most m5C regulatory factors in the two is also different.

### Screening DEGs Between m5C Subtypes and Functional Analysis

**FIGURE 4:**
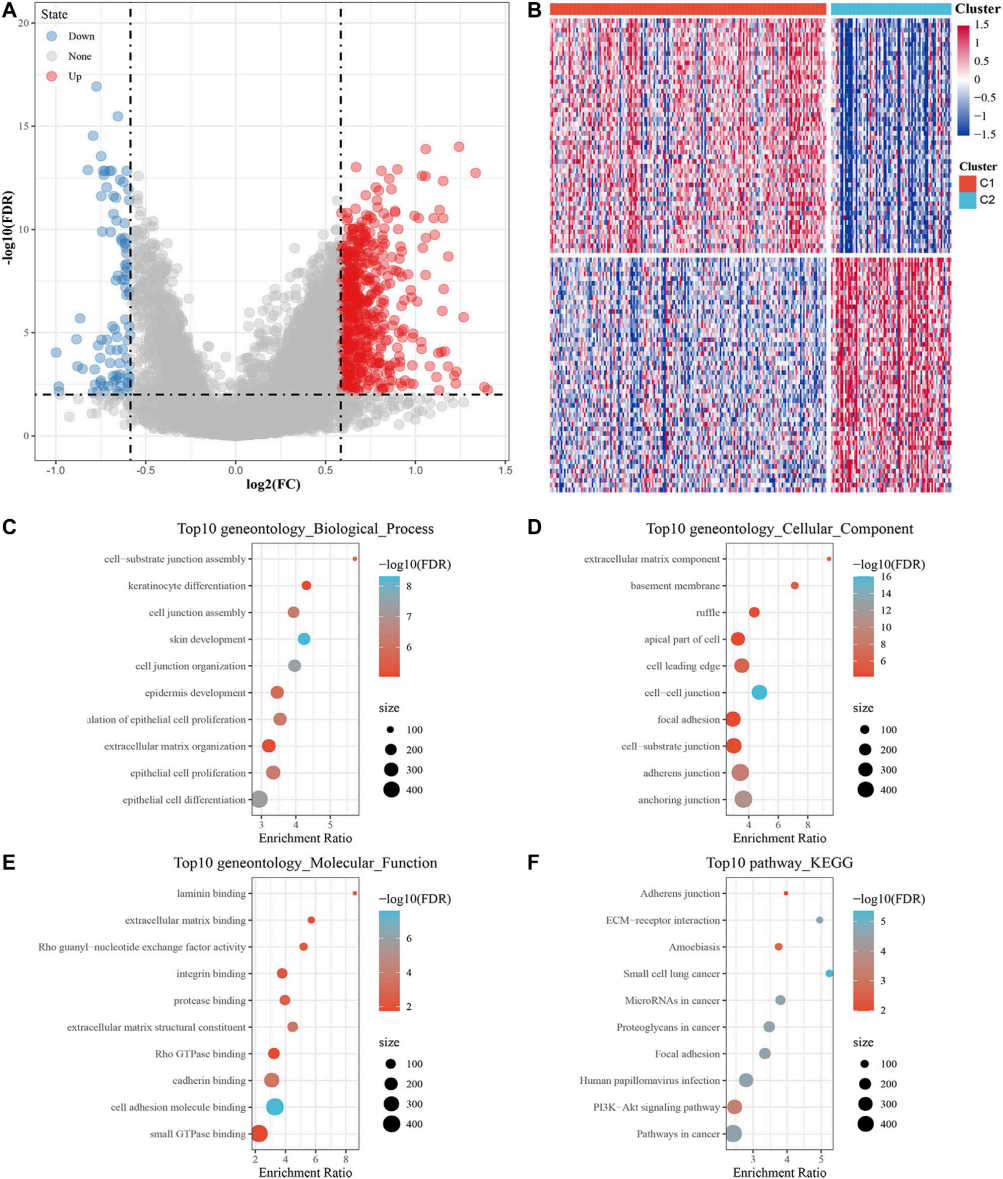
Screening DEGs between m5C subtypes and functional analysis. **(A)** Volcano map of DEGs between the C1 and C2 subtypes. **(B)** Heat map of DEGs between the C1 and C2 subtypes. **(C–E)** The results of GO enrichment analysis of the upregulated DEGs are shown by bubble chart: **(C)** biological processes, **(D)** cell composition and **(E)** molecular function. **(F)** KEGG enrichment analysis of upregulated DEGs.

DEGs between C1 and C2 subtypes consisted of 601 upregulated and 113 downregulated genes. The volcanic map of representative DEGs is represented in Figure 4A. Detailed information about these DEGs is represented in Additional file 2: Supplementary Table S5. We selected 50 genes with the most prominent changes in expression (upregulated and downregulated), as shown in the heat map (Figure 4B).

Next, 601 upregulated DEGs were analyzed by GO function and KEGG pathway enrichment annotation. For the GO function analysis of the upregulated DEGs, 493 gene ontologies were annotated to biological process, 88 to cellular component and 81 to molecular function with significant differences (*p* < 0.05). The top 10 annotations are shown in Figures 4C–E. For the KEGG pathway enrichment analysis of upregulated DEGs, the top 10 KEGG pathways annotated are shown in Figure 4F, including adherens junction, ECM-receptor interaction, amoebiasis, focal adhesion, human papillomavirus infection, PI3K-Akt signaling pathway, and pathways in cancer. More detailed information can be found in Additional file 3: Supplementary Table S6. For CC downregulated DEGs, the results of GO function and KEGG pathway enrichment analysis are shown in Additional file 4: Supplementary Table S7.

### Construction and Evaluation of the Gene Signature in the TCGA Training Set

First, 257 samples in the TCGA-CC dataset were divided into a training set and a test set. The training set consisted of 128 samples, and the test set consisted of 129 samples. The statistical results showed that our groups had no preference, and there was no significant difference between the training set and the test set (Additional file 1: Supplementary Table S2).

**FIGURE 5:**
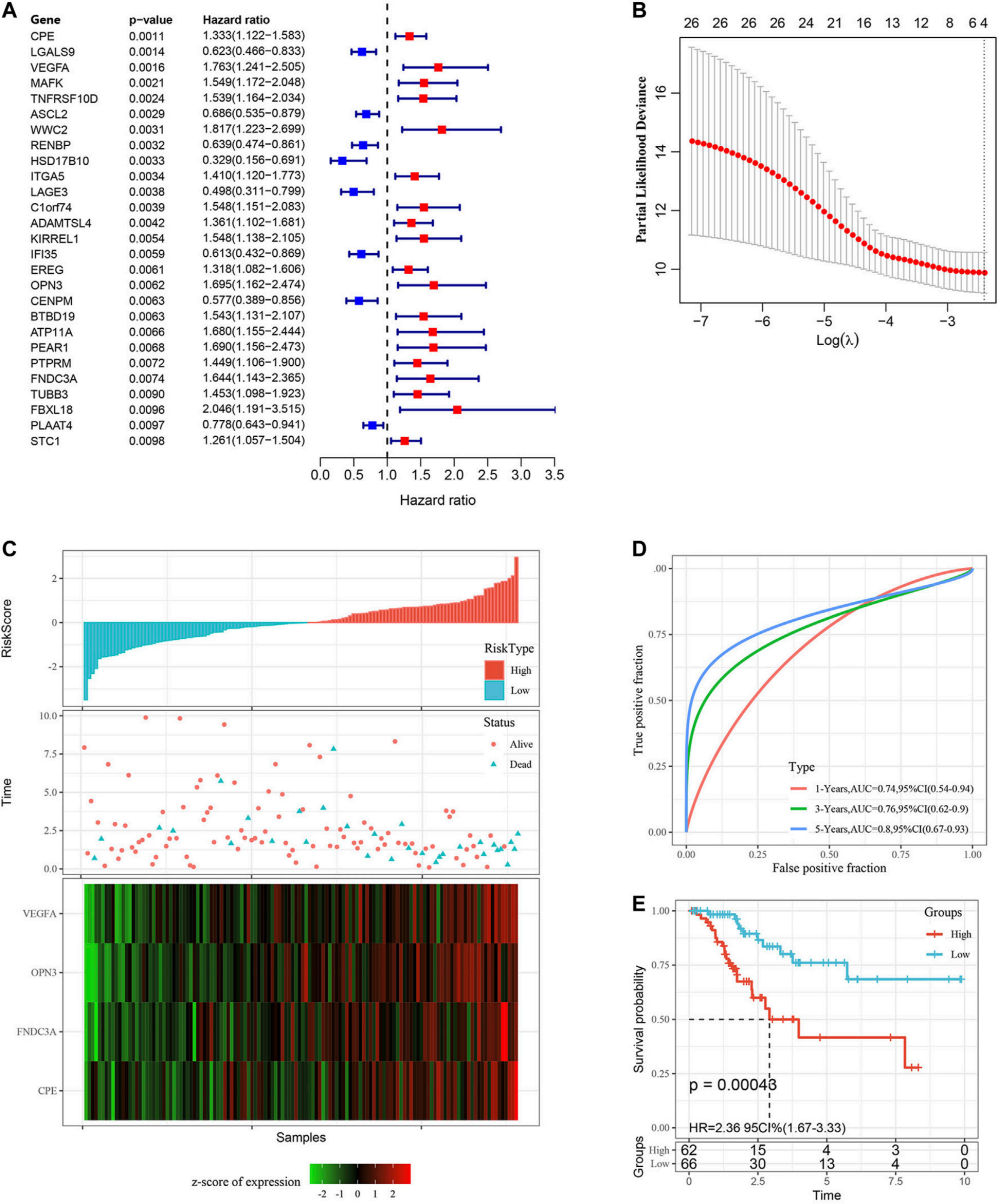
Construction and evaluation of the gene signature in the TCGA training set. **(A)** 27 DEGs were identified by univariate Cox analysis. **(B)** LASSO Cox regression. **(C)** Risk score distribution in the TCGA training set. **(D)** ROC curves were used to assess the efficiency of the risk signature for predicting 1 year, 3 years and 5 years survival rates in the TCGA training set. **(E)** The KM survival curves of the low-risk group and the high-risk group in the TCGA training set. Time: years.

Using the training set data, a univariate Cox proportional hazards regression analysis was conducted by the “survival coxph function” package for DEGs (714 genes) and the survival data, and *p* < 0.01 was selected as the threshold for filtering. Finally, 27 genes were selected, and the univariate Cox analysis results are shown in Figure 5A. Next, we used LASSO regression to further compress these 27 genes to reduce the number of genes in the risk signature. We performed 10-fold cross validation to construct the model and analyzed the confidence intervals under each lambda, as shown in Figure 5B. We selected the following four final genes with lambda = 0.09,009,939: FNDC3A, VEGFA, OPN3 and CPE.

The KM curves all showed that a higher mRNA level of those four genes indicated worse prognosis in the TCGA training set (Additional file 5: Supplementary Figure S1, *p* < 0.05). The final 4-gene signature formula was as follows:

Risk score = 0.3,250,335*FNDC3A (mRNA level)＋0.2,821,988*VEGFA (mRNA level)＋0.3,133,706*OPN3(mRNA level)＋0.1,857,458*CPE (mRNA level).

We calculated the risk score of each sample according to the mRNA level of the signature gene in the training set, and the proportion of deaths in the high-risk group was significantly higher than in the low-risk group. This demonstrated that the risk score is a critical prognostic factor. Consequently, with the increase in risk score, the mRNA levels of FNDC3A, VEGFA, OPN3 and CPE were upregulated (Figure 5C). To investigate the diagnostic accuracy of the risk signature, the AUC of the time-dependent receiver operating characteristic (ROC) curves was computed. The AUC values of the signature for predicting 1 year, 3 years and 5 years survival rates were 0.74, 0.76 and 0.80 (Figure 5D). The KM curve suggested that patients with higher risk score had worse prognosis than those with lower risk score (Figure 5E, *p* < 0.001).

### Validation of the 4-Gene Signature

To determine the robustness of the model, we used the TCGA test set (*n* = 129) with the same model and coefficient as the training set to calculate the risk score of each sample and drew the risk score distribution of the patients. Then, we performed ROC analysis on the prognostic classification of the risk score and analyzed the classification efficiency of 1 year, 3 years and 5 years survival rates. Finally, we divided the samples with risk score into high-risk group (*n* = 65) and low-risk group (*n* = 64) to perform the KM survival analysis. Importantly, the results of the above analysis were consistent with the performance of the TCGA training set (Additional file 6: Supplementary Figure S2 **).**

**FIGURE 6:**
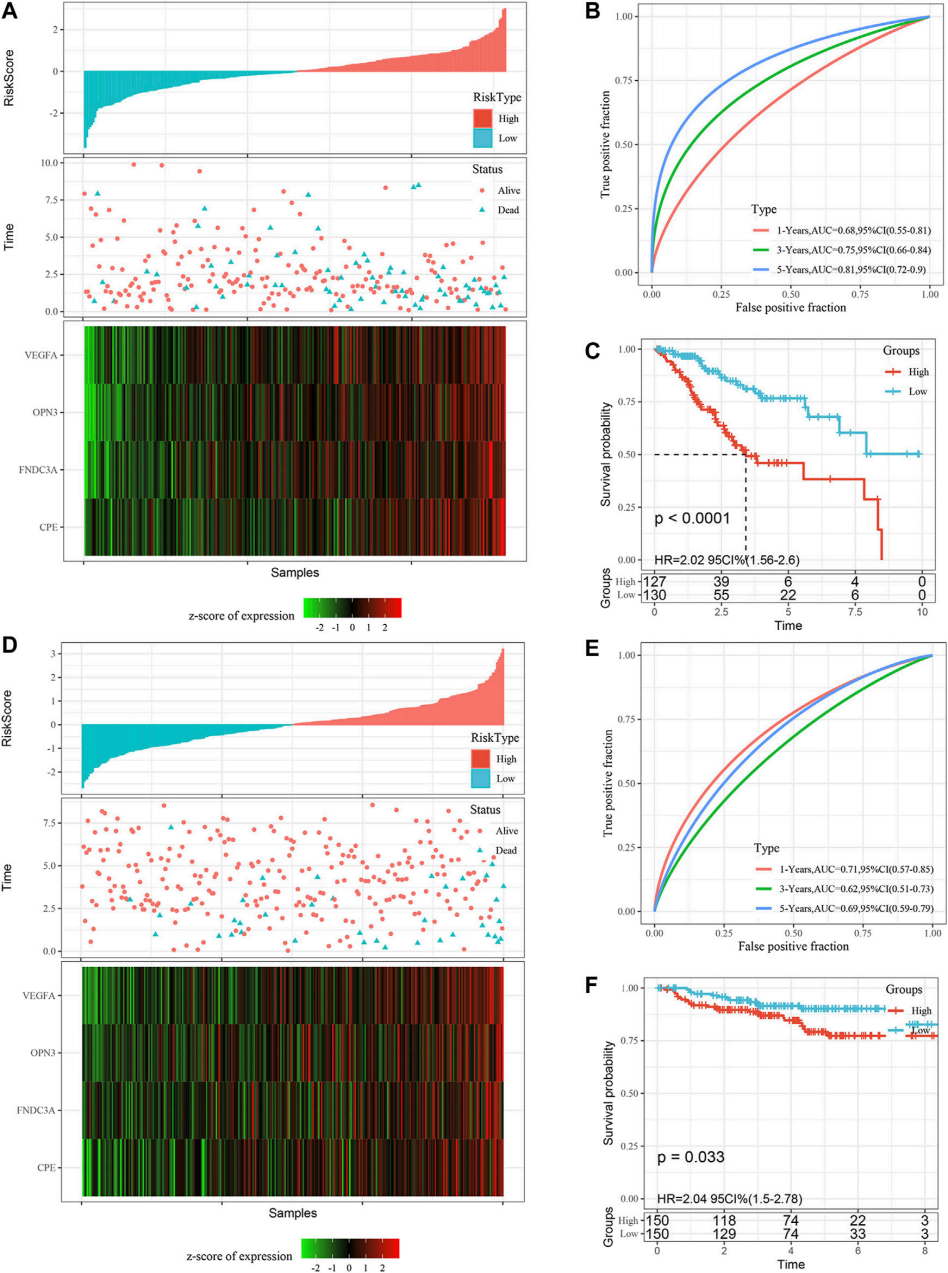
Validation of the gene signature in the TCGA all set and the GSE44001 external validation set. **(A)** Risk score distribution in the TCGA all set. **(B)** ROC curves were used to assess the efficiency of the gene signature for predicting 1 year, 3 years and 5 years survival rates in the TCGA all set. **(C)** The KM survival curves of the low-risk group and the high-risk group in the TCGA all set. **(D)** Risk score distribution in the validation set GSE44001, the survival time of validation set GSE44001 is progression free survival (PFS) time. **(E)** ROC curves were used to assess the efficiency of the gene signature for predicting 1 year, 3 years and 5 years survival rates in the validation set GSE44001. **(F)** The KM survival curves of the low-risk group and the high-risk group in the validation set GSE44001. Time: years.

In addition, we determined the robustness of the signature in the TCGA all set (Figures 6A–C) and the external validation dataset GSE44001 (Figures 6D,E **)**. The proportion of deaths in the high-risk group was significantly higher than in the low-risk group, which was consistent with the performance of the TCGA training set. The AUC values of the signature showed that the risk score was a good prognostic factor. Finally, the KM curve also showed that there were consistent differences between the high-risk and low-risk groups.

### Assessment of the OS Rate of the High-Risk and Low-Risk Groups Based on Different Clinical Subgroups

**FIGURE 7:**
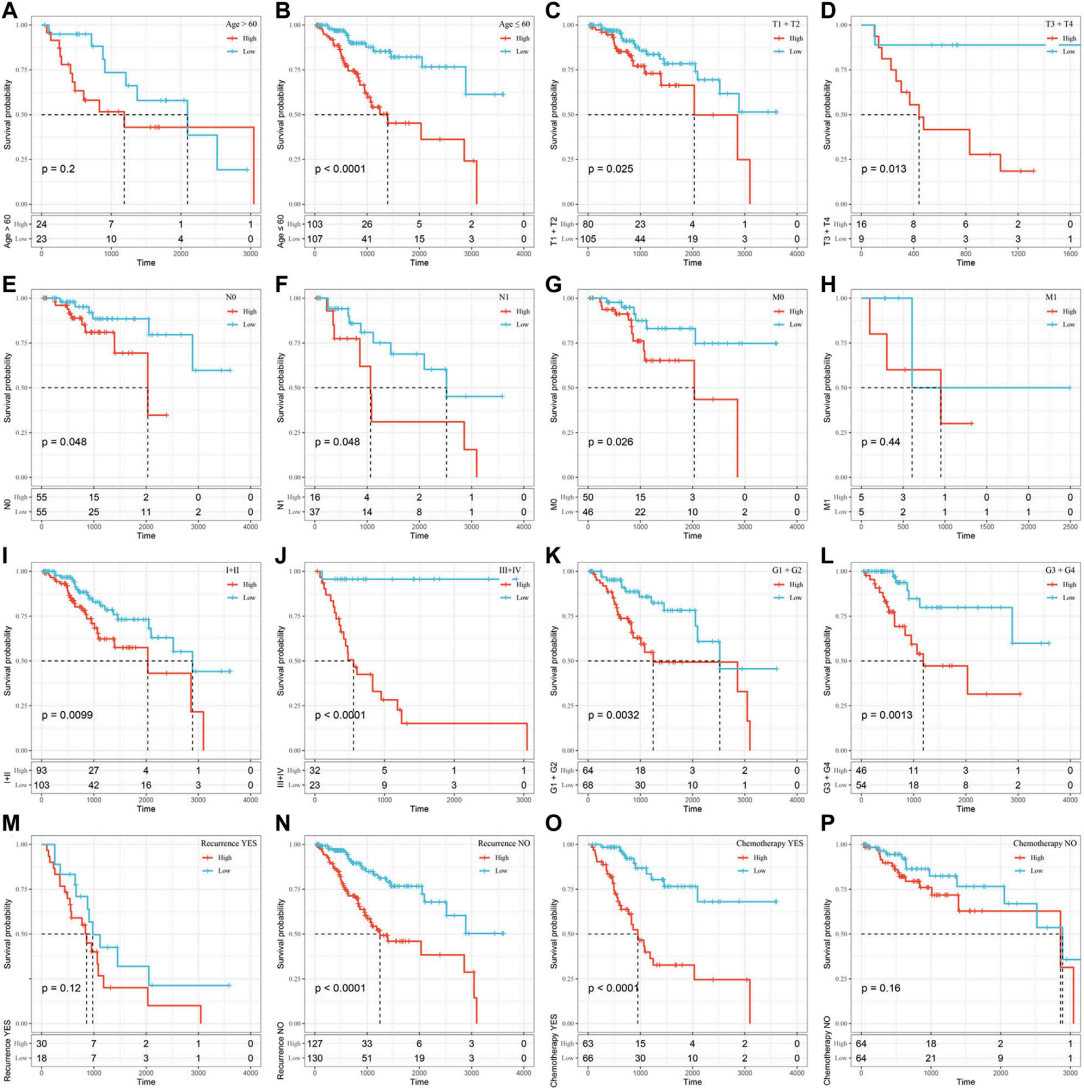
Assessment of the OS rate of the high-risk and low-risk groups based on different clinical subgroups. **(A)** Age>60 subgroup. **(B)** Age≤60 subgroup. **(C)** T1+T2 stage subgroup. **(D)** T3+T4 Stage subgroup. **(E)** N0 Stage subgroup. **(F)** N1 Stage subgroup. **(G)** M0 Stage subgroup. **(H)** M1 Stage subgroup. **(I)**Ⅰ+Ⅱ Stage subgroup. **(J)** Ⅲ+Ⅳ Stage subgroup. **(K)** G1+G2 subgroup. **(L)** G3+G4 subgroup. **(M)** Recurrence_ Yes subgroup. **(N)** Recurrence_No subgroup. **(O)** Chemotherapy _Yes subgroup. **(P)** Chemotherapy_No subgroup. Time: days.

Furthermore, we performed KM survival analysis according to age, grade, stage, recurrence and chemotherapy treatment in the TCGA all set. For the patients in age < = 60, T1+T2 Stage, T3+T4 Stage, N0 Stage, N1 Stage, M0 Stage,Ⅰ+Ⅱ Stage, Ⅲ+Ⅳ Stage, G1+G2, G3+G4, Recurrence _ No and Chemotherapy _ Yes subgroup, the OS interval of patients in the high-risk group was significantly shorter than that of patients in the low-risk group (*p* < 0.05). Only in the age >60, M1, Recurrence _ Yes or Chemotherapy _ No subgroup was the OS interval of patients not different between the high- and low-risk groups (*p* > 0.05). The above findings further showed that the risk signature still has good predictive ability among different clinical subgroups (Figure 7 **)**.

### The Relationship Between Risk Score and KEGG Pathway

**FIGURE 8:**
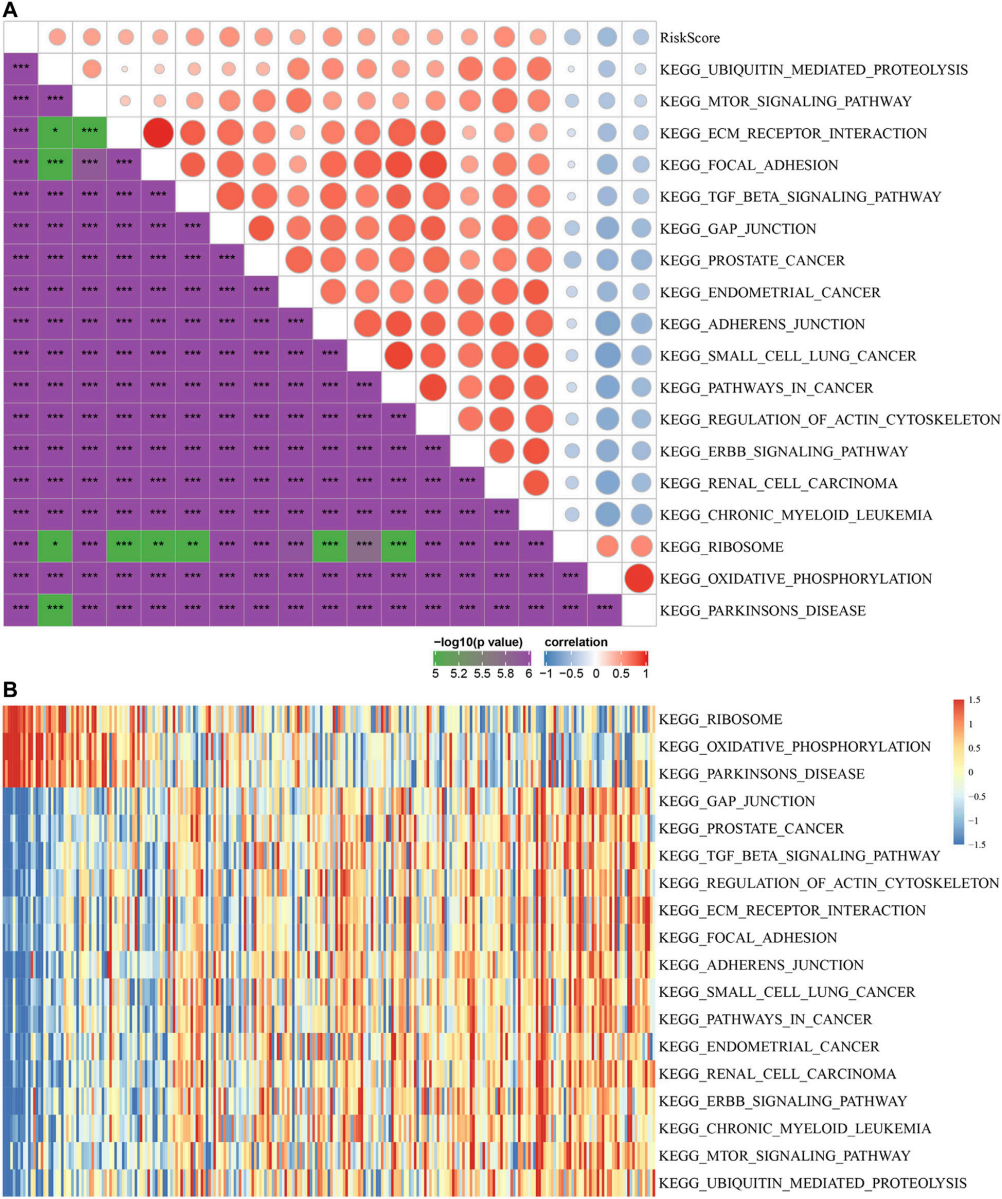
The relationship between risk score and KEGG pathways. **(A)** Correlation coefficient clustering between KEGG pathways and risk score with a risk score correlation greater than 0.4. **(B)** GSVA revealed KEGG pathways associated with the risk score. The horizontal axis represents the sample, and the risk score increases from left to right.

To observe the relationship between risk score and the KEGG pathway, we selected the gene expression profiles corresponding to these samples for ssGSEA using the “GSVA” R package. The score of each sample in different KEGG pathway were calculated, and the ssGSEA score of each KEGG pathway corresponding to each sample were obtained. The correlation between these pathways and risk score was further calculated, and the function with correlation greater than 0.4 was selected (Figure 8A). Fifteen pathways were positively correlated with the risk score, and three pathways were negatively correlated with the risk score. The top 18 KEGG pathways were selected and clustered according to their enrichment score, as shown in Figure 8B. We can find that KEGG_MTOR_SIGNALING_PATHWAY, KEGG_ECM_RECEPTOR_INTERACTION, KEGG_FOCAL_ADHESION, KEGG _TGF_BETA_SIGNALING_PATHWAY, KEGG_ADHERENS_JUNCTION, KEGG_ PATHWAYS_IN_CANCER and other tumor-related pathways were activated with increasing risk score.

### Construction and Evaluation of a Nomogram

**FIGURE 9:**
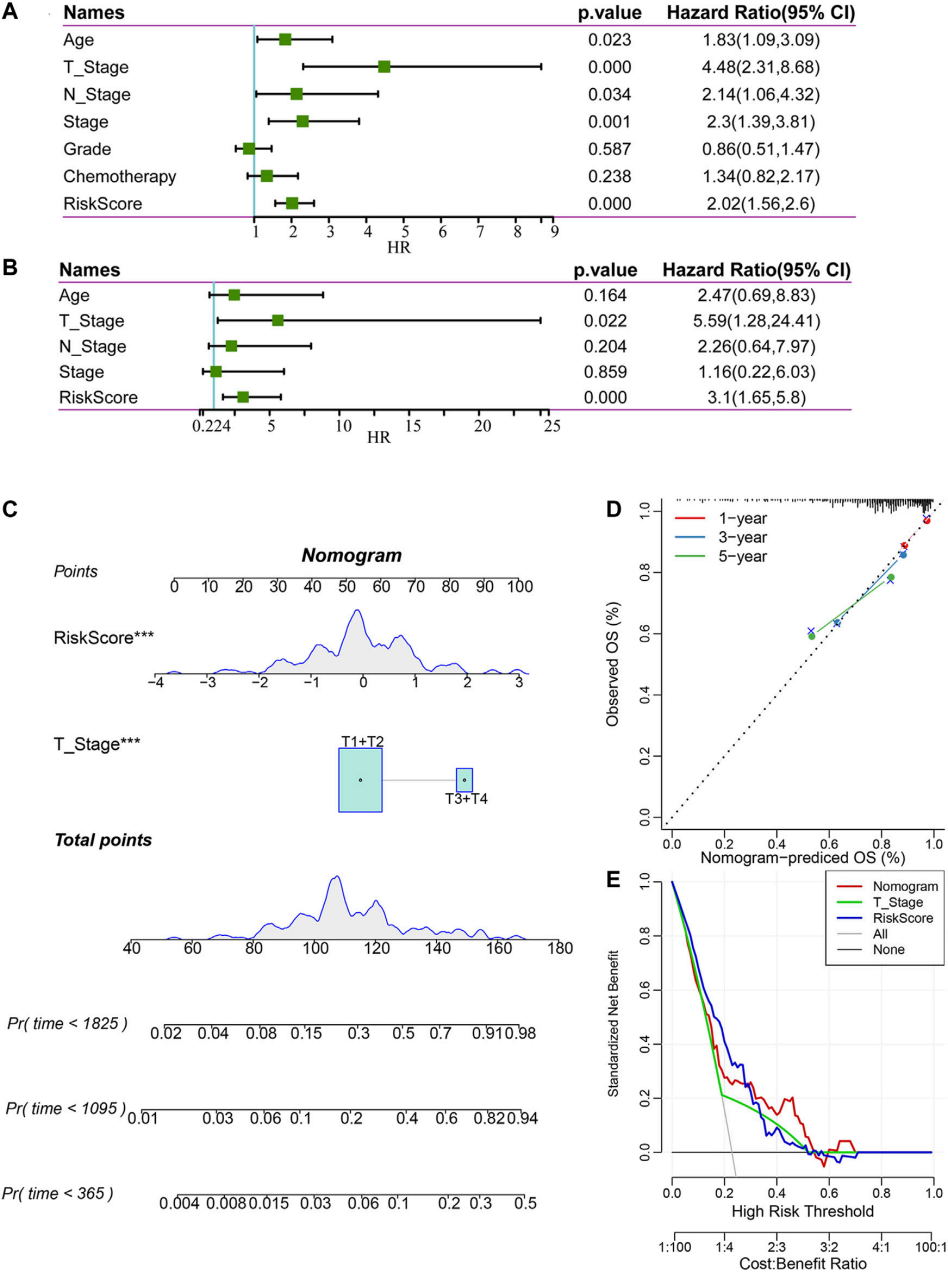
Construction and evaluation of a nomogram. **(A)** Univariate Cox regression analysis of clinical characteristics and risk score. **(B)** Multivariate Cox analysis of clinical characteristics and risk score. **(C)** A nomogram for predicting the 1 year, 3 years and 5 years survival rates of CC patients was established. **(D)** 1 year, 3 years, and 5 years survival rate calibration curves of the line chart. **(E)** The DCA of the nomogram.

To identify the independence of the 4-gene signature in clinical parameters, we used univariate and multivariate Cox regression to analyze the related HR, 95% CI of HR and *p* value in the clinical information of the TCGA all set. The clinical data of patients were analyzed systematically, including age, T stage, N stage, FIGO stage, grade, chemotherapy and risk score (Figures 9A,B). In the TCGA all set, the risk score was an independent prognostic factor. Therefore, the 4-gene signature has good predictive performance and clinical application value.

According to the results of univariate and multivariate Cox regression analyses, we constructed a nomogram with clinical features, T stage and risk score (Figure 9C). We found that the risk score had the greatest impact on survival prediction, indicating that the risk score is indispensable in the nomogram. Furthermore, we used the calibration curve to evaluate the prediction accuracy of the signature, as shown in Figure 9D. The prediction calibration curves of the three calibration points for 1 year, 3 years and 5 years survival rates were close to the standard curves, indicating that the signature had good prediction performance. In addition, DCA showed that the benefits of the risk score and nomogram were significantly higher than those of the extreme curves. The nomogram curve was higher than that of the risk score, which indicated that the nomogram had good reliability (Figure 9E).

### Signature Gene Expression Was Upregulated in Cervical Cancer

**FIGURE 10:**
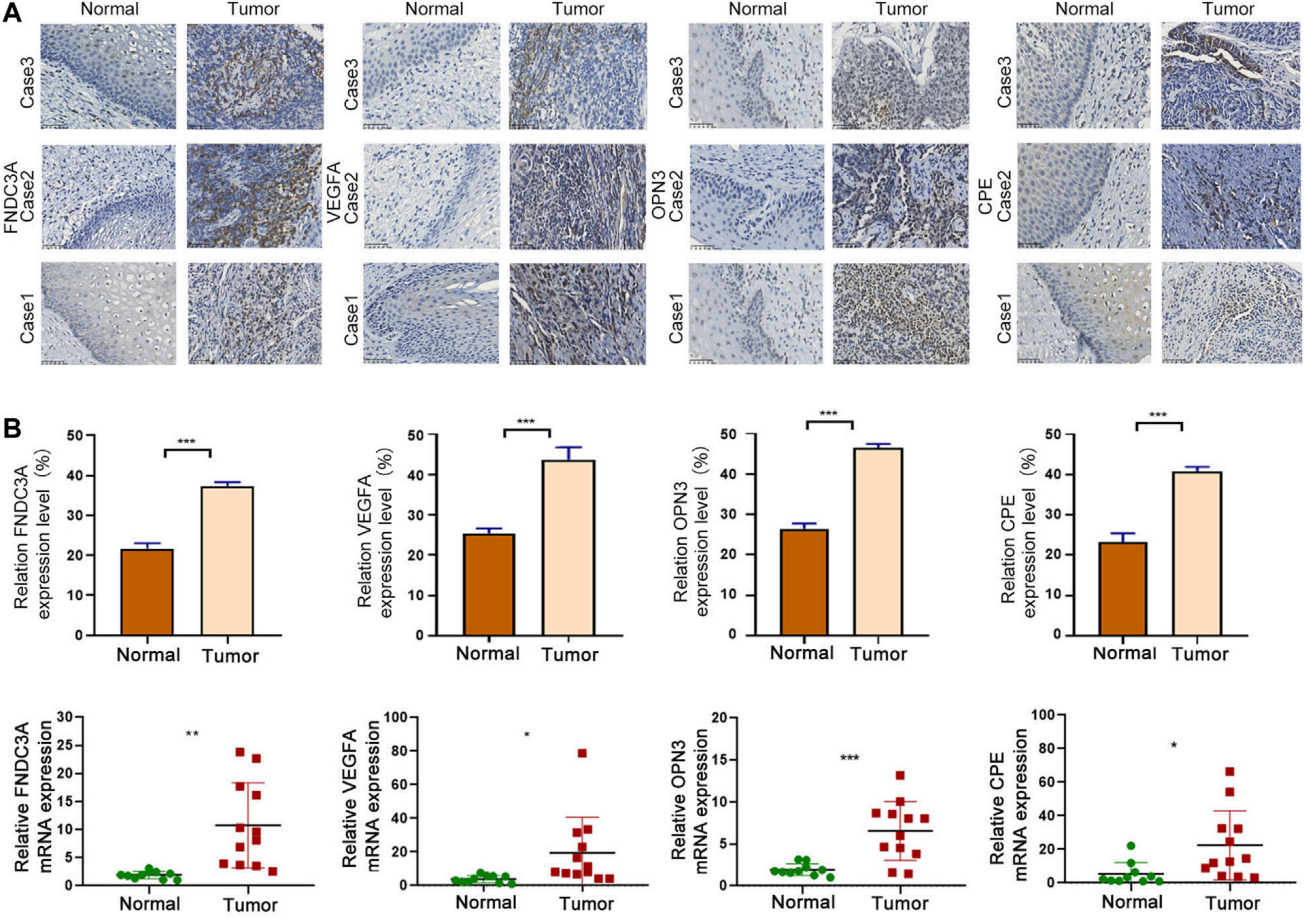
Signature gene expression was upregulated in CC. **(A)** IHC analysis of FNDC3A, VEGFA, OPN3 and CPE protein levels in tumor and normal tissues. Scale bar = 50 μm ***p < 0.001. **(B)** qRT-PCR analysis of FNDC3A, VEGFA, OPN3 and CPE mRNA levels in tumor and normal tissues. *, *p* < 0.05. **, *p* < 0.01. ***, *p* < 0.001.

In order to explore the difference in protein expression of signature genes, we used IHC to detect 6 normal and 21 tumor tissues. The IHC quantization analysis was calculated by ImageJ software and statistically analyzed in three random fields. The results showed that the protein expression levels of the four signature genes in tumor were higher than those in normal (Figure 10A). At the same time, we conducted qRT-PCR experiments in 10 normal and 12 tumor tissues to explore the differences in the transcription level of those four genes. It showed that the mRNA levels of the four model genes were significantly increased in tumor tissues, indicating that the expression of model genes in tumor tissues may be abnormally activated (Figure 10B).

### FNDC3A, VEGFA or CPE Promoted the Proliferation, Invasion and Migration of SiHa Cells

**FIGURE 11:**
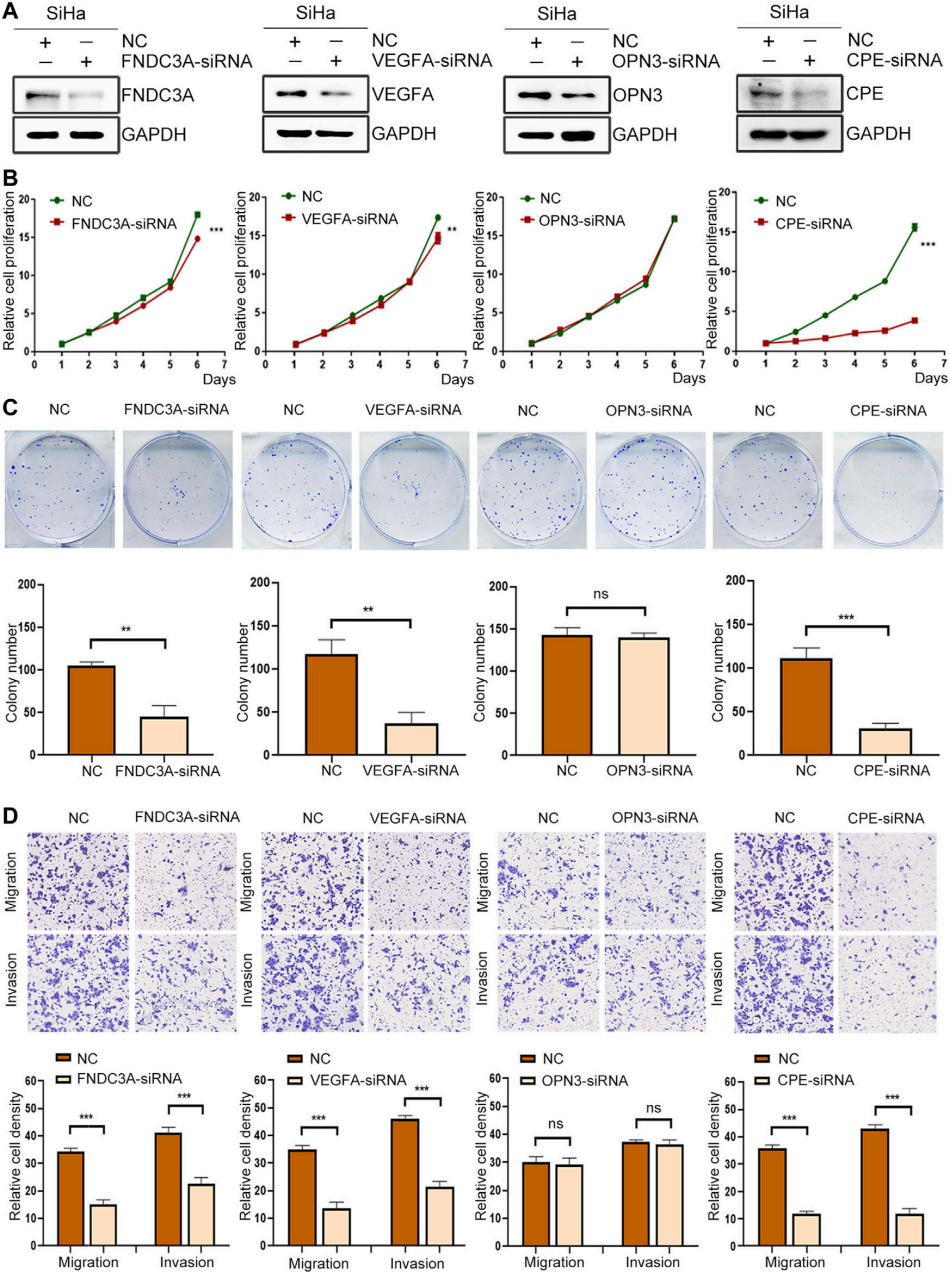
FNDC3A, VEGFA or CPE promoted the proliferation, invasion and migration of SiHa cells. **(A)** Western blotting analysis of FNDC3A, VEGFA, OPN3 or CPE expression in SiHa cells transfected with their siRNA. **(B)** Cell proliferation abilities were detected by CCK-8. **(C)** Colony number were detected. **(D)** Cell migration and invasion abilities were evaluated by Transwell assay. NC: Negative Control. **, *p* < 0.01; ***, *p* < 0.001.

To clarify the functional role of FNDC3A, VEGFA, OPN3 and CPE in CC cells, we applied human-specific siRNA to decrease their protein expression (Figure 11A). The CCK-8 assay was applied to detect cell proliferation. The downregulation of FNDC3A, VEGFA or CPE expression significantly suppressed the proliferation (Figure 11B) and colony formation capacity of SiHa cells (Figure 11C). Transwell assays were applied to detect the invasion and migration ability of SiHa cells *in vitro*, and the number of cells that passed through the polycarbonate membrane was smaller in the FNDC3A, VEGFA or CPE siRNA group than in the negative control group, indicating that FNDC3A, VEGFA or CPE could significantly promote the invasion and migration of SiHa cells (Figure 11D **)**. Downregulation of OPN3 expression had no significant effect on the proliferation, colony formation ability, invasion and migration of SiHa cells.

## Discussion

m5C is common methylation modification in eukaryotic RNA, which can promote the regulation of nuclear mRNA through the methyltransferase NSUN2 and the binding protein ALYREF and participates in the splicing and protein translation process of several mRNAs (Yang et al., 2017). It was reported that m5C methylation of the 3′-UTR contributes to an increase in mRNA stability (Zhang et al., 2012); tRNA occurs most frequently on cytosine of the variable arm and C38 of the anticodon ring (Agris, 2008; Trixl and Lusser, 2019), which can maintain the thermal stability of the tRNA secondary structure and improve the recognition ability of codons. Besides, the rRNA of all organisms is modified by m5C, methylation sites of human and yeast 28S rRNA, M5C2870 and M5C2278, are critical for protein translation (Sharma and Lafontaine, 2015). In addition, modification of m5C can also be detected in noncoding RNAs, such as lncRNAs, erRNAs, and vtRNAs (Amort et al., 2013; David et al., 2017).

m5C modification plays an important role in the development of tumors, such as that of NSUN family proteins (RNA m5C methyltransferase). Compared with normal human tissues and cells, the expression of NSUN2 is increased in a variety of tumor tissues, and NSUN2 is considered to be an effective prognostic marker for some cancers, such as squamous cell carcinomas and colon carcinomas (Frye and Watt, 2006). In breast cancer cells, NSUN6 can form a complex with the proteins LLGL2 and lncRNA Maya, which inactivates the kinase Hippo/MST1 through methylation of Hippo/MST1, resulting in promotion of tumor metastasis (Li et al., 2017). However, the role and mechanism of m5C RNA modification in the prognosis of CC have not been studied.

We hypothesized that m5C RNA modification-related genes have broad prospects in the prognostic evaluation of CC. First, we extracted the mRNA levels of 13 m5C regulatory factors from the TCGA expression matrix for clustering and obtained two subtypes of CC, C1 and C2. Next, we identified the differences in the immune infiltration levels between the two molecular subtypes. We screened DEGs between the C1 and C2 subtypes and obtained 601 upregulated genes and 113 downregulated genes. Next, we divided the TCGA all set (257 samples) into a training set and a test set. In the TCGA training set, we used univariate Cox regression and LASSO regression analysis to establish a 4-gene signature comprising FNDC3A, VEGFA, OPN3 and CPE. Then, we conducted risk distribution analysis, ROC, curve analysis and survival analysis in the TCGA training set, the TCGA test set, the TCGA all set and the GSE44001 data set to verify our signature. Furthermore, we found that in most clinical feature subgroups, the OS interval of patients in the high-risk group was significantly shorter than that of patients in the low-risk group (*p* < 0.05). In addition, we proved that the risk score was an independent risk factor and constructed an effective nomogram to predict the 1 year, 3 years and 5 years survival rates of CC patients.

To explore the function of signature genes and their prognostic correlation in CC patients, we carried out qRT-PCR and IHC experiments and found that the mRNA levels and protein expression of those genes were all higher in cervical cancer tissues than in normal tissues. In addition, downregulation of FNDC3A, VEGFA or CPE expression suppressed the proliferation, migration and invasion of SiHa cells *in vitro*. KM survival analysis showed that high expression of the four hub genes was a risk factor for CC patients. FNDC3A can be used as a prognostic marker for colorectal cancer and is highly expressed in colon cancer tissues, and high expression of FNDC3A increases the mortality rate of colon cancer patients (Meyer et al., 2012; Wuensch et al., 2019). In multiple myeloma, high expression of FNDC3A can lead to ROS accumulation, ATP deficiency and cell death in multiple myeloma cells. VEGFA can be used as a prognostic biomarker for head and neck squamous cell carcinoma, esophageal squamous cell carcinoma, glioblastoma and papillary thyroid carcinoma (He et al., 2020; Stuchi et al., 2020; Yang et al., 2020; Zheng and Tao, 2020). Downregulation of VEGFA expression can inhibit the proliferation, angiogenesis and metastasis of osteosarcoma cells, ovarian cancer and lung squamous cell carcinoma (Chen et al., 2020a; Chen et al., 2020b; Li et al., 2020; Qin et al., 2020). Upregulation of VEGFA expression can promote the proliferation, angiogenesis and metastasis of gastric cancer cells and breast cancer cells (Chen et al., 2020a; Wang et al., 2020). OPN3 can be used as a prognostic biomarker for lung adenocarcinoma. With the increase in OPN3 expression, the mortality rate of lung adenocarcinoma patients increases, and the survival time decreases (Wang et al., 2019). It has been reported that the OPN3 gene enhances the metastasis of lung adenocarcinoma, and its overexpression promotes epithelial-mesenchymal transition (Xu et al., 2020). In lung carcinoids, patients with high OPN3 expression are more likely to experience relapse and metastasis (Miyanaga et al., 2020). In addition, OPN3 can also sensitize liver cancer cells to 5-fluorouracil treatment by regulating the apoptosis pathway (Jiao et al., 2012). In a recent study, CPE was used to predict recurrence of early lung adenocarcinoma (Jones et al., 2021). In addition, CPE expression was upregulated in patients with extranasal nodal natural killer cell/T cell lymphoma (NKTCL) after cytarabine chemotherapy and could be used as a chemotherapy index for NKTCL patients (Gong et al., 2018).

In summary, we developed a novel 4-gene signature based on m5c modification, which had good AUC in the training set and three validation sets. Based on the signature, we constructed an effective nomogram to predict the 1 year, 3 years and 5 years survival rates of CC patients. We suggested using this classifier as a molecular diagnostic test to evaluate the prognostic risk of CC patients. Furthermore, we found that three of the signature genes (FNDC3A, VEGFA or CPE) function as oncogenes to promote the proliferation, invasion and migration of cervical cancer cells and could be potential therapeutic targets for CC. The advantage of this study is that we identified a prognostic 4-gene signature with a relatively high AUC in the training and three validation datasets, which can accurately predict survival rates. Then we explored the expression and function of the signature genes to explore the potential of these genes as therapeutic targets. The limitation of this study is that we should further carry out animal experiments to verify the function of model genes, in addition, the molecular mechanisms of model genes regulating the progression of CC still need to be further explored.

## Data Availability

The original contributions presented in the study are included in the article/Supplementary Material, further inquiries can be directed to the corresponding authors.

## Abbreviations

CC, cervical cancer; m5C: 5-methylcytidine; DEGs, differentially expressed genes; GO, Gene Ontology; KEGG, Kyoto Encyclopedia of Genes and Genomes; TCGA, The Cancer Genome Atlas; ROC, receiver operating characteristic; HR, hazard ratio; CI, confidence interval; OS, overall survival; PFS, progression free survival; GSVA, Gene set variation analysis; IHC, Immunohistochemistry; NMF, nonnegative matrix factorization; KM, Kaplan-Meier; HPV, Human papillomavirus; GEO, Gene Expression Omnibus; ssGSEA, single sample Gene set enrichment analysis; LASSO, least absolute shrinkage and selection operator; DCA, decision curve analysis; BP, biological processes; CC, cell composition; MF, molecular function.
