# Supplementary material for: Development and Validation of a Novel Gene Signature for Predicting the Prognosis by Identifying m5C Modification Subtypes of Cervical Cancer
Source: Front Genet. 2021 Sep 22;12:733715. doi: 10.3389/fgene.2021.733715 (PMC8493221; doi:10.3389/fgene.2021.733715)
Supplement: Supplementary file 1 [file Presentation1.zip › Table 1-4.DOCX]

**Additional file 1:**

**Table S1: TCGA all set sample information table**

| Clinical Features | TCGA all set |
| --- | --- |
| **Status** |  |
| 0 | 188 |
| 1 | 69 |
| **T Stage** |  |
| T1 | 121 |
| T2 | 64 |
| T3 | 15 |
| T4 | 10 |
| Tis | 1 |
| TX | 16 |
| Missing value | 30 |
| **N Stage** |  |
| N0 | 110 |
| N1 | 53 |
| NX | 64 |
| Missing value | 30 |
| **M Stage** |  |
| M0 | 96 |
| M1 | 10 |
| MX | 119 |
| Missing value | 32 |
| **Stage** |  |
| I | 138 |
| II | 58 |
| III | 36 |
| IV | 19 |
| NA | 6 |
| **Grade** |  |
| G1 | 16 |
| G2 | 116 |
| G3 | 99 |
| G4 | 1 |
| NA | 25 |
| **Age** |  |
| ≤60 | 210 |
| >60 | 47 |

**Table S2:** **TCGA training set and TCGA test set sample information table**

| Clinical Features | TCGA-training set | TCGA-test set | P |
| --- | --- | --- | --- |
| **Status** |  |  |  |
| 0 | 96 | 92 | 0.5739 |
| 1 | 32 | 37 |  |
| T Stage |  |  |  |
| T1 | 65 | 56 | 0.3219 |
| T2 | 31 | 33 |  |
| T3 | 9 | 6 |  |
| T4 | 4 | 6 |  |
| Tis | 1 | 0 |  |
| TX | 4 | 12 |  |
| Missing value | 14 | 16 |  |
| N Stage |  |  |  |
| N0 | 55 | 55 | 0.0588 |
| N1 | 34 | 19 |  |
| NX | 25 | 39 |  |
| Missing value | 14 | 16 |  |
| M Stage |  |  |  |
| M0 | 49 | 47 | 0.2533 |
| M1 | 8 | 2 |  |
| MX | 56 | 63 |  |
| Missing value | 15 | 17 |  |
| Stage |  |  |  |
| I | 74 | 64 | 0.6531 |
| II | 28 | 30 |  |
| III | 15 | 21 |  |
| IV | 9 | 10 |  |
| NA | 2 | 4 |  |
| Grade |  |  |  |
| G1 | 11 | 5 | 0.0873 |
| G2 | 55 | 61 |  |
| G3 | 54 | 45 |  |
| G4 | 0 | 1 |  |
| NA | 8 | 17 |  |
| Age |  |  |  |
| ≤60 | 108 | 102 | 0.3332 |
| >60 | 20 | 27 |  |

**Table S3.** **Gene siRNA sequence**

| Gene | Sequence of siRNA（5’ to 3’） |
| --- | --- |
| FNDC3A | ATGAGTCAACATCCTATAAAT |
| VEGFA | AGGGCAGAATCATCACGAAGT |
| OPN3 | GGTCTGTTGGATGCCTTATAT |
| CPE | CTCCAGGCTATCTGGCAATAA |

**Table S4.** **Primers sequence for qRT-PCR analysis**

| Gene | Primer | Sequence（5’ to 3’） |
| --- | --- | --- |
| GAPDH | Forward | CAGCCTCAAGATCATCAGCA |
|  | Reverse | TGTGGTCATGAGTCCTTCCA |
| FNDC3A | Forward | GCACAAGTGAATTGGGAGGT |
|  | Reverse | CACAACACTCAGAGCCTGGA |
| VEGFA | Forward | CCTTGCTGCTCTACCTCCAC |
|  | Reverse | ATCTGCATGGTGATGTTGGA |
| OPN3 | Forward | CTGGAGGGCCATTACCTACA |
|  | Reverse | TCCTTGGATTTCCAGTCCAC |
| CPE | Forward | GTACCTGGAGGGATGCAAGA |
|  | Reverse | TGAAGGTCTCGGACAAATCC |
