# Supplementary figures and images for: Development and Validation of a Novel Gene Signature for Predicting the Prognosis by Identifying m5C Modification Subtypes of Cervical Cancer
Source: Front Genet. 2021 Sep 22;12:733715. doi: 10.3389/fgene.2021.733715 (PMC8493221; doi:10.3389/fgene.2021.733715)

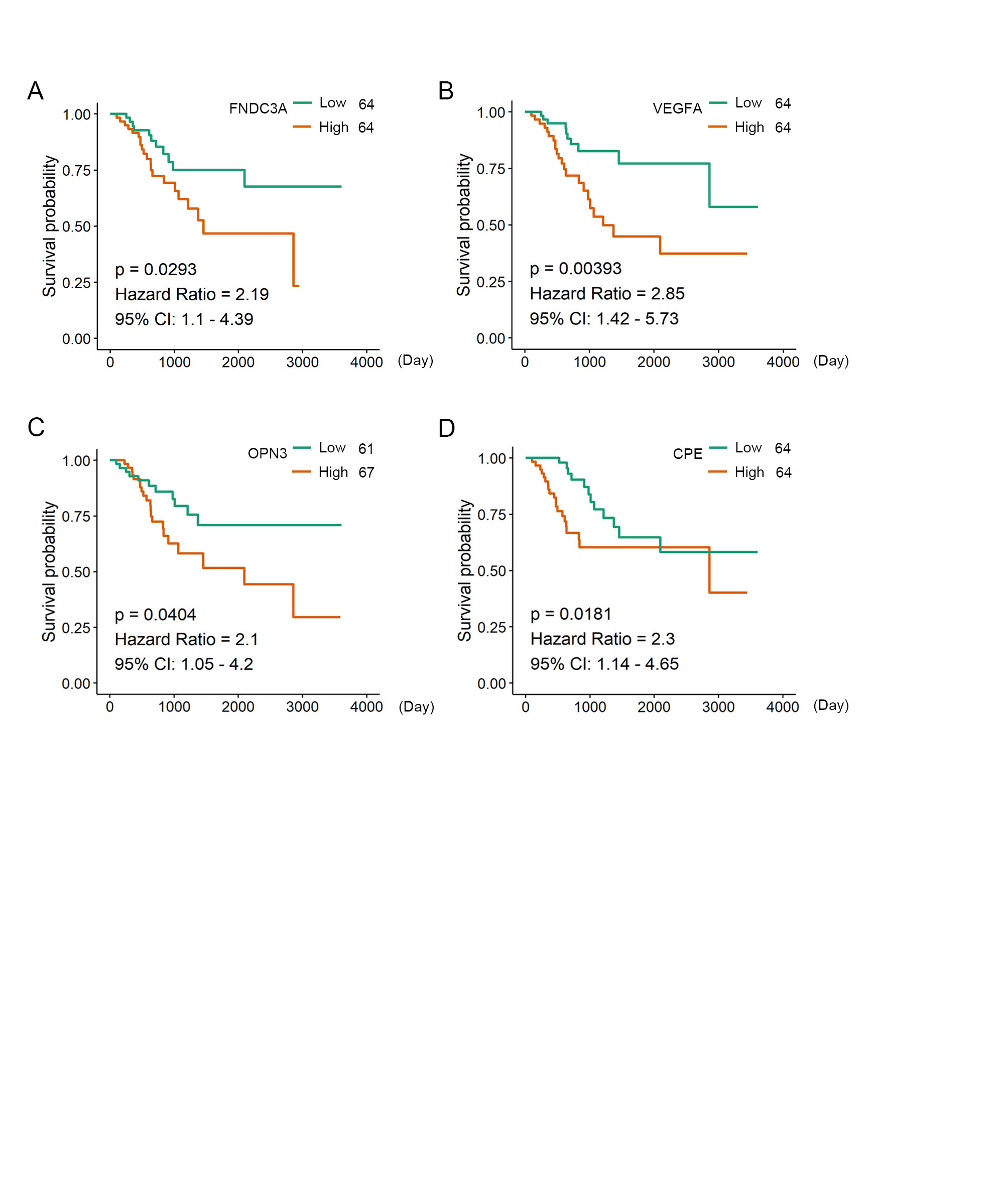

Supplement: Supplementary file 2 [file Image1.JPEG]

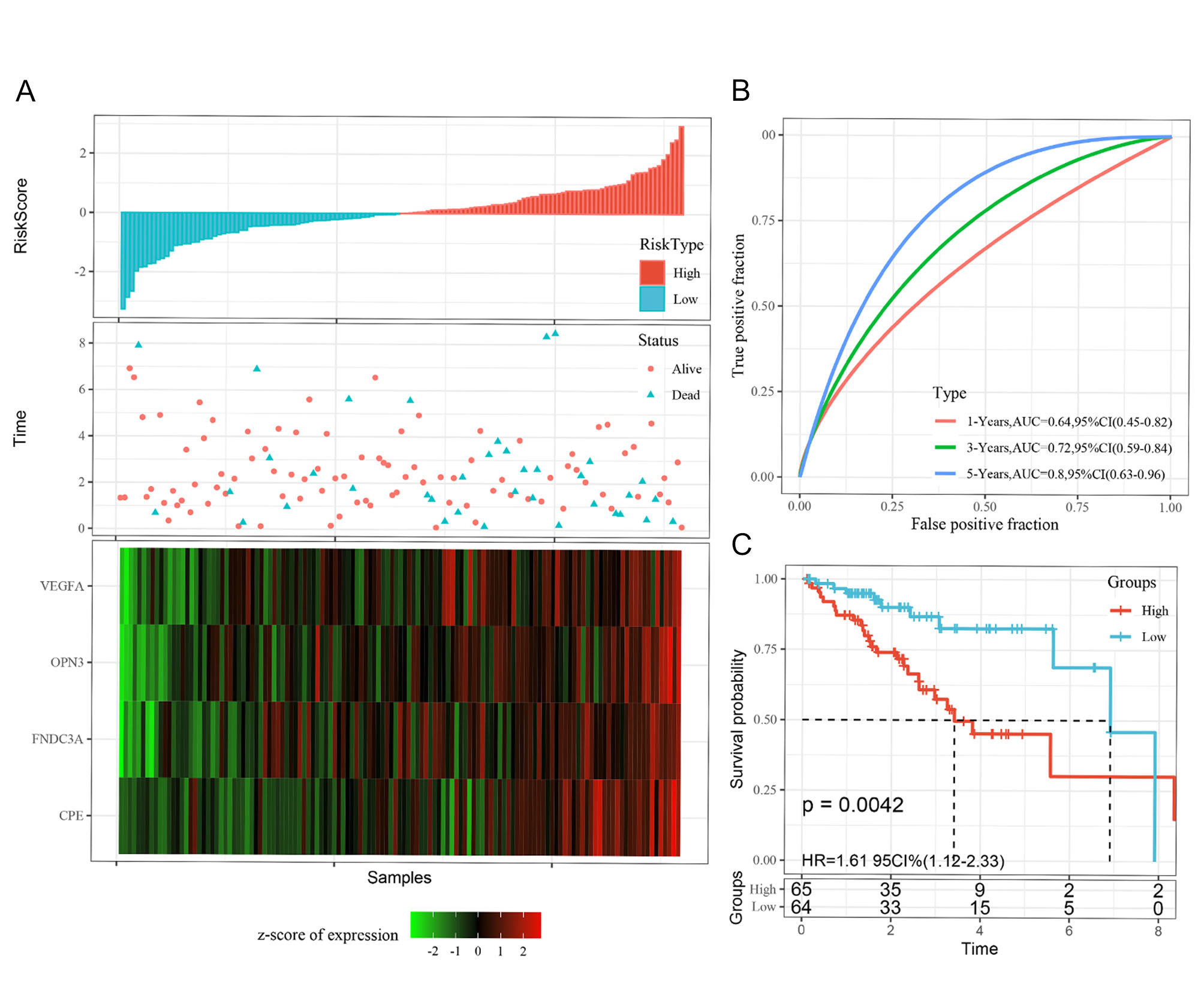

Supplement: Supplementary file 3 [file Image2.JPEG]
